# Validation of the perceived personal responsibility and desire for reconciliation scales in the Spanish population

**DOI:** 10.1371/journal.pone.0336599

**Published:** 2025-11-13

**Authors:** Karla Gallo-Giunzioni, Agata Kasprzak, María Prieto-Ursúa, Cristina Fernández-Belinchón

**Affiliations:** 1 Faculty of Education and Psychology, Universidad Francisco de Vitoria, Madrid, Spain; 2 Instituto del Perdón, Universidad Francisco de Vitoria, Madrid, Spain; 3 Department of Psychology, Faculty of Social and Human Sciences, Universidad Pontificia Comillas, Madrid, Spain; 4 Servicio de Urgencias Médicas de Madrid (SUMMA 112), Madrid, Spain; King Khalid University, EGYPT

## Abstract

**Introduction:**

Perceived personal responsibility and the desire for reconciliation are crucial in the study of the forgiveness process; however, there are few instruments for measuring these variables that have been adapted and validated for the Spanish population. Therefore the aim of the present study is to validate the Perceived Personal Responsibility Scale and the Desire for Reconciliation Scale for the Spanish population and evaluate their psychometric properties.

**Method:**

Sample composed of 499 participants (181 men, 318 women) aged from 18 to 67 years (M = 38.35; SD = 13.44). After the translation and linguistic adaptation of the scales, the Exploratory Factor Analysis (EFA) was conducted with subsample 1 and the Confirmatory Factor Analysis (CFA) with subsample 2.

**Results:**

The EFA identified one factor for both the Perceived Personal Responsibility Scale and the Desire for Reconciliation Scale. The CFA confirmed a good fit for the unifactorial model for the Perceived Personal Responsibility Scale (CFI = .978, TLI = .955, IFI = .978, RMSEA = .079) and a good fit for the unifactorial model for the Desire for Reconciliation Scale (CFI = .984, TLI = .951, IFI = .981, RMSEA = .064). The reliability of the Perceived Personal Responsibility Scale ranged from.80 to.84, and the reliability of the Desire for Reconciliation Scale ranged from.84 to.88.

**Discussion:**

The results of this study show that both the Perceived Personal Responsibility Scale and the Desire for Reconciliation Scale are reliable and useful instruments for application in Spain. With these measures, it will be possible to further study processes such as self-forgiveness, which is constantly growing in the Spanish population. In addition, the study also provides measures that are easy to apply in clinical practice.

## Introduction

Forgiveness has emerged as a crucial topic in contemporary psychological research due to its relevance to personal well-being, interpersonal relationships, and social harmony. In particular, self-forgiveness has gained increasing attention as a complex intrapersonal process with implications for mental health, personal growth, and the restoration of moral identity [1–4]. In a societal context marked by conflict, polarization, and a growing interest in emotional and relational health, the ability to forgive oneself—when genuinely warranted—has become a central aspect of psychological functioning and intervention [3–7].

However, self-forgiveness is not a simple process of letting go of guilt or improving one’s self-image. On the contrary, it involves confronting one’s own wrongdoing with honesty and integrity. Theoretical frameworks emphasize that authentic self-forgiveness requires two key components: the assumption of personal responsibility for the offense and the desire to repair the damage done, often reflected in the desire for reconciliation with the victim or the community [1,8,9]. These two constructs—personal responsibility and reconciliation—have been proposed as markers that distinguish genuine self-forgiveness from defensive strategies such as self-justification or denial [2,5,8,10,11].

Perceived personal responsibility has been extensively studied as a foundational element of self-forgiveness. Enright and his collaborators defined self-forgiveness as “the desire to abandon resentment toward oneself for a wrong done, while fostering compassion, generosity, and love toward oneself” [12, p. 115]. This process presupposes acknowledging having caused harm and a moral commitment to face the consequences. Accepting responsibility means an increase in social threats that humans tend to avoid, such as the social exclusion faced by offenders as a consequence of their actions [13], being psychologically uncomfortable and painful, and often accompanied by feelings of guilt, shame, and discomfort [13–15].

Without accepting responsibility, true self-forgiveness does not occur [10], and any attempt at forgiveness risks becoming pseudo self-forgiveness—a psychological mechanism through which individuals avoid guilt without genuine moral repair [8,10,13]. This defensive pattern implies that the offender engages in cognitive restructuring of their offense to reduce the negative emotions derived from their wrongful actions, and may involve denying the offense, minimizing the harm caused, or attributing blame to others, thus obstructing the possibility of true reconciliation or personal growth [8–10,13].

In this regard, Fisher and Exline [14] proposed the Perceived Personal Responsibility Scale as a brief measure to distinguish genuine self-forgiveness from mere excusing of the self. Despite its widespread use in English-speaking contexts, this instrument has not been adapted or validated for the Spanish population. This lack of adaptation limits its applicability and accuracy in Spanish-speaking contexts, as cultural, linguistic, and social differences can influence how individuals interpret and respond to the constructs measured by the instrument.

The second crucial construct is the desire for reconciliation, understood as the offender’s willingness to restore a damaged relationship or make reparations to the victim. The most recent literature on self-forgiveness underscores that genuine self-forgiveness is not only an intrapersonal process but also includes interpersonal dimensions [2,16], specially empathy and willingness to reconcile with their victims [9]. Forgiving oneself leads to reparative behaviors with the environment, the offending situation and the offended; indeed, some authors understand that it would only be appropriate for the offender to forgive himself or herself if he or she does so after the victim has already forgiven him or her [10].

The most recent research on self-forgiveness insists on the importance of taking responsibility and reparative behaviors towards the victim as prerequisites for self-forgiveness [17]. For Woodyatt and Wenzel [2], only that which includes interpersonal and intrapersonal restoration is true self-forgiveness. True self-forgiveness seems to be achieved through affirmation of the values that have been violated, which generates a greater desire for reconciliation [16]. The presence—or absence—of this desire may therefore serve as an important indicator of the quality and completeness of the self-forgiveness process [2].

To measure this construct, Woodyatt and Wenzel [1] developed the Desire for Reconciliation Scale, a four-item instrument designed to capture the intention to repair interpersonal relationships following an offense. Similar to the Perceived Personal Responsibility Scale, this measure has not yet been validated in Spanish, nor have any cultural adaptations been identified. Given the growing interest in self-forgiveness research and its clinical applications, it is essential to have validated tools for assessing these constructs within Spanish-speaking populations. In this context, the selection of the Spanish population as a case study is not solely driven by the need to translate and adapt the instrument into Spanish or to broaden the geographical scope of the research. Rather, it is based on the fact that this population shares common traits with other cultures at an international level, while also exhibiting unique cultural characteristics [18,19]. This combination makes the Spanish population a particularly suitable context for in-depth analysis of the processes involved in self-forgiveness, thereby generating relevant insights that may be applicable across diverse cultural settings.

Therefore, the aim of this study is to validate the Spanish versions of two instruments: the Perceived Personal Responsibility Scale [14] and the Desire for Reconciliation Scale [1], by analyzing their psychometric properties in a heterogeneous sample. These measures will allow for a more accurate and culturally appropriate assessment of self-forgiveness processes and their associated dimensions in both research and applied settings in Spain.

## Materials and methods

### Participants

In this study, a convenience sample of 499 participants was used, consisting of 181 men (36.7%) and 318 women (63.7%) aged between 18 and 67 years (M = 38.35; SD = 13.44). All participants were Spanish nationals and Spanish speakers. Regarding marital status: 47.3% married or in a domestic partnership, 45.7% single, 6% divorced and 1% widowed. Regarding educational level: 4% primary education, 18.4% secondary education, 37.1% higher education (degree/license) and 39.9% postgraduate or doctoral studies.

### Measures

*Sociodemographic Questionnaire.* Created for the purposes of this research, it collected data on age, sex, nationality, marital status and completed studies of the participants.

*Perceived Personal Responsibility Scale* [14]. This instrument consists of five items, including both direct statements such as “I feel guilty for what I did” and indirect ones like “I really did nothing wrong”. Each item is answered on a 10-point Likert scale (1 = completely disagree to 10 = completely agree). High scores indicate greater perceived self-responsibility. The authors report an adequate Cronbach’s alpha (.83) [14].

*Desire for Reconciliation Scale* [1]*.* This instrument evaluates the desire for reconciliation of those who have committed an offense through four items. These include statements like “I want to reconcile with this person” and “I want the relationship between this person and me to improve.” Participants respond on a 7-point Likert scale (1 = completely disagree to 7 = completely agree). High scores on the Desire for Reconciliation scale indicate a greater intention to repair the relationship with the offended person. Woodyatt & Wenzel [1] provide evidence of adequate reliability (.82).

*Differentiated Self-Forgiveness Process Scale* [2]*.* This instrument consists of 20 items that measure levels of genuine self-forgiveness, self-punishment, and pseudo-self-forgiveness. High scores on the subscales indicate higher levels in each measured variable. Participants respond on a 7-point Likert scale. Items 1–7 comprise the Genuine Self-Forgiveness subscale, items 8–14 form the Self-Punishment subscale, and items 15–20 correspond to the Pseudo-Self-Forgiveness or Self-Exoneration subscale. Woodyatt and Wenzel [2] report adequate reliability for each subscale: Genuine Self-Forgiveness (.85), Pseudo-Self-Forgiveness (.81), and Self-Condemnation (.85). In the present study, similar values were obtained, with a Cronbach’s alpha of.85 for the Genuine Self-Forgiveness subscale,.77 for the Pseudo-Self-Forgiveness scale and.81 for the Self-Condemnation subscale.

### Procedure

The study followed the guidelines of the International Test Commission for the translation and cross-cultural adaptation of instruments [20,21]. The scales used were subjected to a direct and reverse translation process to ensure that the Spanish version was equivalent to the English version. The original English scales were translated into Spanish and independently reviewed by three bilingual psychologists. After comparing the three versions, which were very similar, a single Spanish version was agreed upon. From this version, two other bilingual individuals were asked to perform a reverse translation into English. Finally, after verifying their correspondence with the original scales, the final version of the scales was established and administered to the participants (see S1 and S2 Appendices) along with informed consent and the other described instruments. The data collection began in February 2023 and concluded in July of the same year.

This research was part of a doctoral thesis, which was previously reviewed and approved by the Academic Doctoral Committee of Universidad Pontificia Comillas. Subsequently, it did not require approval from the ethics committee. Participants signed an online informed consent form in accordance with the current European regulations on personal data protection.

### Data analysis

To analyzing for this study, the sample was randomly divided into two subsamples: the first (n = 149) for Exploratory Factor Analysis (EFA) and the second (n = 350) for Confirmatory Factor Analysis (CFA). Tables 1 and 2 presents the descriptive analysis of the items.

**Table 1:** Descriptive data of the items (n = 149).

		*M*	*SD*	Skewness	Kurtosis
Reconciliation Desire Scale	DR 1	6.44	1.21	−2.35	5.06
	DR 2	5.93	1.77	−1.66	1.66
	DR 3	5.79	1.87	−1.44	0.84
	DR 4	5.25	2.19	−0.91	−0.68
Perceived Personal Responsibility Scale	RPP 1	6.66	2.59	−0.68	−0.37
	RPP 2	6.19	2.71	−0.28	−0.86
	RPP 3	6.69	2.88	−0.55	−0.83
	RPP 4	5.35	2.78	0.13	−1.04
	RPP 5	6.72	2.61	−0.61	−0.43

*M = *mean*; SD =* standard deviation; DR- Desire for Reconciliation Scale, RPP- Perceived Personal Responsibility Scale.

**Table 2:** Descriptive data of the items (n = 350).

		*M*	*SD*	Skewness	Kurtosis
Desire for Reconciliation Scale	DR 1	6.49	1.06	−2.75	8.76
	DR 2	6.02	1.67	−1.70	1.92
	DR 3	5.95	1.74	−1.71	1.86
	DR 4	5.42	2.05	−1.01	−0.36
Perceived Personal Responsibility Scale	RPP 1	6.57	2.70	−0.48	−0.73
	RPP 2	5.99	2.79	−0.23	−1.00
	RPP 3	6.79	2.82	−0.58	−0.78
	RPP 4	5.42	2.93	0.01	−1.16
	RPP 5	6.34	2.92	−0.36	−1.07

*M = *mean*; SD =* standard deviation; DR- Desire for Reconciliation Scale, RPP- Perceived Personal Responsibility Scale

To analyze the behavior of the Perceived Personal Responsibility Scale by Fisher and Exline [14] and the Desire for Reconciliation Scale by Woodyatt and Wenzel in a Spanish sample, the EFA was performed using version 25 of the SPSS program. For the sample size required to conduct CFA, it is recommended to have sufficiently large samples. Authors like Nunnally and Bernstein [22] recommend having at least 10 participants per item, this study met this requirement (n = 149).

Prior to performing the EFA, a descriptive analysis of the items of both scales was conducted using univariate and multivariate tests such as the Kolmogorov-Smirnov and Mardia’s test.

To verify the adequacy of the data for EFA, the Kaiser-Meyer-Olkin (KMO) measure and Bartlett’s test of sphericity were reviewed. Once the data’s adequacy for EFA was confirmed, following recommendations from authors such as Fabrigar et al. [23] and O’Connor [24], the number of factors was estimated using the parallel analysis using the Generalized Least Squares method. For the EFA, due to the characteristics of the data and the inability to assume univariate normality (as assessed by the Kolmogorov-Smirnov test) and multivariate normality (as assessed by Mardia’s test), the Generalized Least Squares extraction method was used for both scales. This decision was based on evidence that when Likert-type scales include 5–7 categories, it is reasonable to approximate them as continuous, and GLS can be considered an acceptable option in exploratory analyses under moderate violations of normality [25].

To confirm the factorial structure of the Perceived Responsibility Scale and the Desire for Reconciliation Scale, CFA was performed using the Jamovi program version 2.3.18. Following the recommendations by Finney and DiStefano [26] and Fidell et al. [27] for CFA on estimation methods for non-normal data the Maximum Likelihood Robust Estimation (MLR) method was used. MLR was chosen because it adjusts both standard errors and fit indices against non-normality and has been shown to perform comparably to WLSMV with Likert data of 5–7 categories [28–32].

The chi-squared and indices of the goodness of fit (RMSEA, CFI, TLI, IFI and NFI) of the data to the model were analyzed.

Reliability was calculated using Cronbach’s alpha, following the values recommended by George and Mallery [33], where α ≥ .5 is considered poor, > .6 questionable, > .7 acceptable, > .8 good, and >.9 excellent.

Criterion validity was analyzed through Spearman correlations between the Perceived Personal Responsibility Scale and Desire for Reconciliation Scale and the subscales of the Differentiated Self-Forgiveness Process. These analyses were conducted using version 25.0 of the SPSS statistical program.

## Results

### Exploratory factor analysis

#### Adequacy of the data for exploratory factor analysis.

For the Desire for Reconciliation Scale, the Kaiser-Meyer-Olkin (KMO) measure of sampling adequacy was adequate with a value of KMO = .77. Bartlett’s test of sphericity yielded χ² = 426.69; gl = 6; p = .000. The parallel analysis results (Table 3) identified a single factor.

**Table 3:** Parallel analysis of the desire for reconciliation scale.

Factors	Empirical eigenvalues	Random eigenvalues	Eigenvalues 95% percentile
1	2.67*	0.20	0.32
2	0.09	0.06	0.13
3	−0.07	−0.04	0.01
4	−0.09	−0.14	−0.08

* The empirical eigenvalue is larger than the random eigenvalue.

For the Perceived Personal Responsibility Scale, the KMO measure was adequate with a value of KMO = .76. Bartlett’s test of sphericity yielded χ² = 235.927; df = 10; p = .000. Using the Kaiser-Gutman rule, an initial single-factor solution was obtained, explaining 55.35% of the total variance. Similarly, parallel analysis results (Table 4) identified a single factor.

**Table 4:** Parallel analysis of the perceived personal responsibility scale.

Factors	Empirical eigenvalues	Random eigenvalues	Eigenvalues 95% percentile
1	2.23*	0.26	0.39
2	0.26	0.11	0.21
3	−0.07	0.01	0.07
4	−0.14	−0.08	−0.02
5	−0.22	−0.18	−0.12

* The empirical eigenvalue is larger than the random eigenvalue.

#### Exploratory factor analysis.

Table 5 presents the factor loadings for each scale. As can be seen, all factor loadings exceed.45, the recommended criterion by authors such as Tabachnick & Fidell [34]. The results indicate an unifactorial structure for both scales, consistent with the original authors’ structure.

**Table 5:** Exploratory factor analysis of the desire for reconciliation scale and the perceived personal responsibility scale: Factor matrix.

Item	Factor
DR 1	.54
DR 2	.94
DR 3	.95
DR 4	.80
PPR 1	.80
PPR 2	.65
PPR 3	.60
PPR 4	.82
PPR 5	.49

DR = Desire for Reconciliation Scale; PPR = Perceived Personal Responsibility Scale.

#### Reliability.

Regarding the reliability of the scales studied, Cronbach’s alpha for the Desire for Reconciliation Scale was.88, and for the Perceived Personal Responsibility Scale, it was.80.

#### Confirmatory factor analysis.

**Fig 1:**
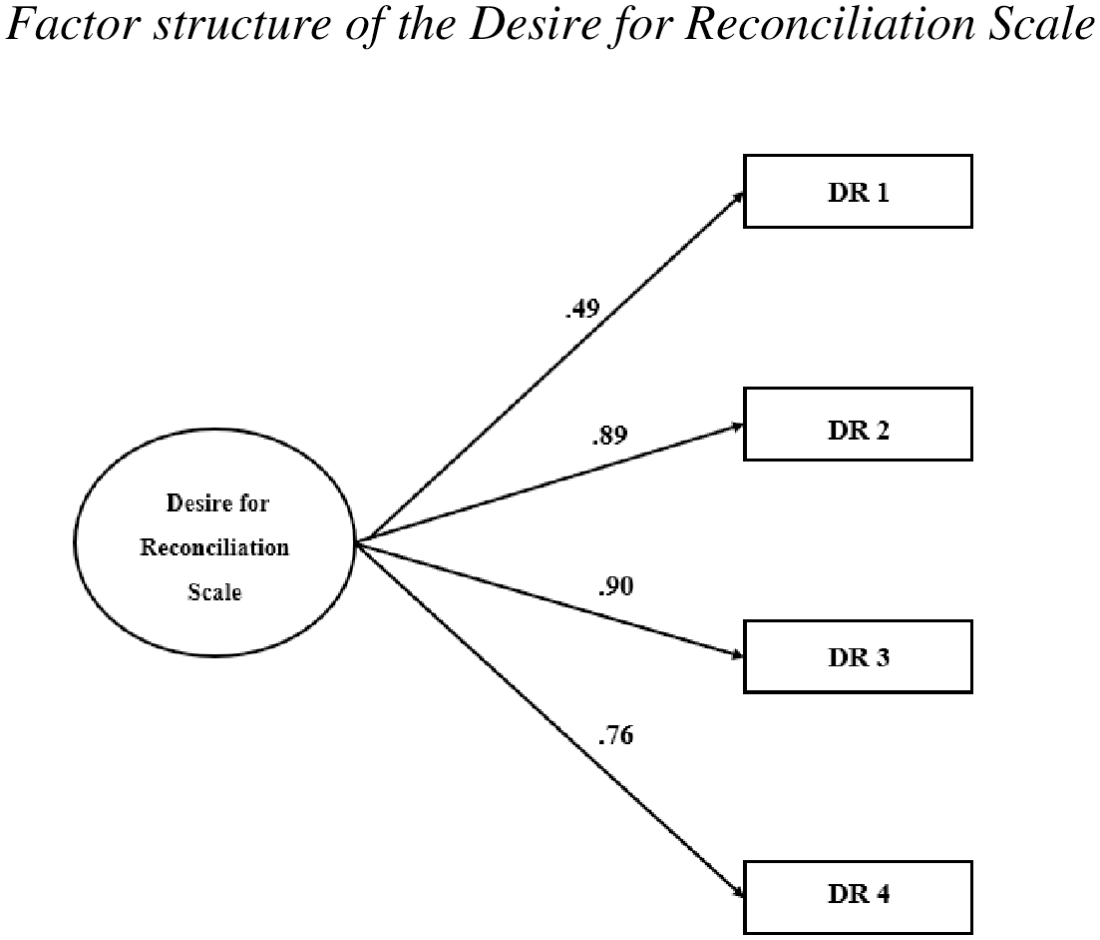
Factor structure of the desire for reconciliation scale.

For the Desire for Reconciliation Scale, the CFA results for the unifactorial model proposed by Woodyatt and Wenzel [2] indicated a good model fit to the Spanish sample. As shown in Table 6, the RMSEA value was.064, and the model fit indices (CFI, TLI, IFI, and NFI) had scores above.95. The factor loading range for the model is between.49 (item 1) and.90 (item 3) as shown in Fig 1.

**Table 6:** Indices of goodness of fit of the desire for reconciliation scale.

χ²	gl	p	CFI	TLI	IFI	NFI	RMSEA (IC 95)
29.82	26	< .001	.984	.951	.984	.981	.064 (.020−.108)

**Fig 2:**
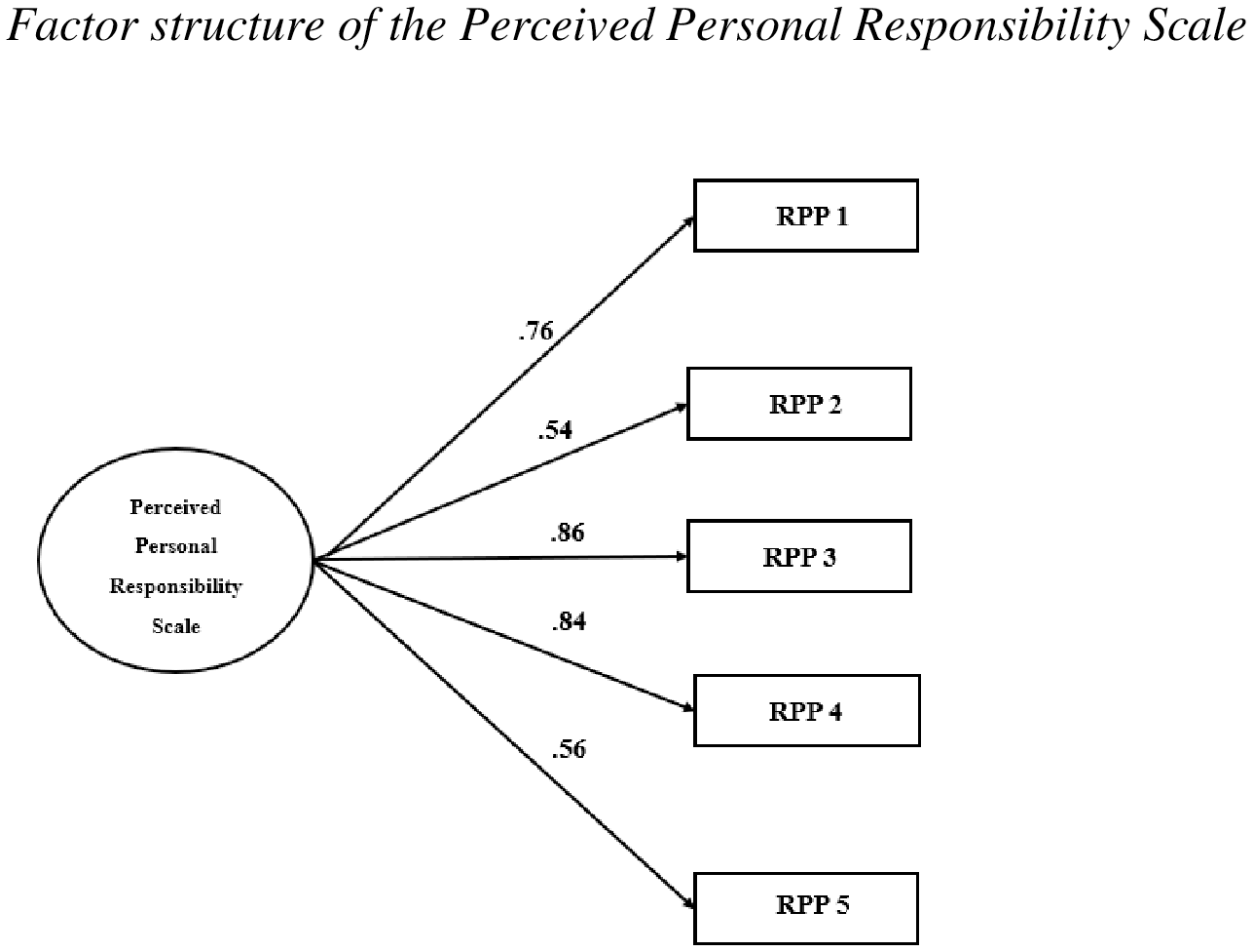
Factor structure of the perceived personal responsibility scale.

For the Perceived Personal Responsibility Scale, the CFA results for the unifactorial model proposed by Fisher and Exline [14] also indicated a good model fit to the Spanish sample. As shown in Table 7, the RMSEA value was.079, and the model fit indices (CFI, TLI, IFI, and NFI) had scores above.95. The factor loading range for the model is between.54 (item 2) and.86 (item 3) as shown in Fig 2.

**Table 7:** Indices of goodness of fit of the perceived personal responsibility scale.

χ²	gl	p	CFI	TLI	IFI	NFI	RMSEA (IC 95)
58.21	10	< .001	.978	.955	.978	.971	.079 (.042−.118)

The RMSEA value for both scales is below the recommended.10 value by Weston and Gore [35]. For CFI, the value for both scales is above.95, indicating good model fit according to Lai [36]. For TLI, the value is above the.90 cutoff recommended by Xia and Yang [37]. Lastly, the IFI and NFI scores are above.95, aligning with the values recommended by various authors [38–40].

#### Reliability.

Regarding the reliability of the studied scales, Cronbach’s alpha for the Desire for Reconciliation Scale was.84, and for the Perceived Personal Responsibility Scale, it was.84.

#### Criterion validity.

Regarding the criterion validity of the Desire for Reconciliation Scale, it was analyzed through Spearman correlations between the total score of the Reconciliation Desire Scale and the scores of the Genuine Self-Forgiveness, Pseudo Self-Forgiveness, and Self-Condemnation subscales. As shown in Table 8, a significant but weak positive relationship was found between the total score of the Desire for Reconciliation Scale and the scores of the Genuine Self-Forgiveness and Self-Condemnation subscales. Similarly, a weak negative relationship was found between the total score of the Reconciliation Desire Scale and the Pseudo Self-Forgiveness subscale.

**Table 8:** Correlations between desire for reconciliation, perceived personal responsibility, genuine self-forgiveness, self-condemnation, and pseudo self-forgiveness.

	SFG	SC	PSF
Desire for Reconciliation Scale	.26**	.17**	−.23**
Perceived Personal Responsibility Scale	.43**	.48**	−.68**

** **p* *< .01; SFG- Genuine Self-Forgiveness; SC- Self-Condemnation; PSF- Pseudo Self-Forgiveness; DR- Desire for Reconciliation Scale, RPP- Perceived Personal Responsibility Scale.

On the other hand, the criterion validity of the Perceived Personal Responsibility Scale was analyzed through Spearman correlations between the total score of the Perceived Personal Responsibility Scale and the scores of the Genuine Self-Forgiveness, Pseudo Self-Forgiveness, and Self-Condemnation subscales.

As shown in Table 8, a significant high positive relationship was found between the total score of the Perceived Personal Responsibility Scale and the scores of the Genuine Self-Forgiveness and Self-Condemnation subscales. Additionally, a significant strong negative relationship was found between the total score of the Perceived Personal Responsibility Scale and the Pseudo Self-Forgiveness subscale.

## Discussion

The present study provides empirical evidence supporting the validity and reliability of the Spanish versions of two widely used instruments: the Perceived Personal Responsibility Scale and the Desire for Reconciliation Scale. These findings confirm the unifactorial structure and adequate internal consistency of both scales in a Spanish-speaking sample, thereby facilitating their application in research and clinical settings in Spanish cultural contexts.

Regarding the Perceived Personal Responsibility Scale by Fisher and Exline [14], the analyses conducted in this study provide significant evidence about its psychometric properties, especially in relation to its dimensional structure previously not analyzed by the original authors. Through EFA, the presence of a single factor was identified, suggesting that the items on this scale are highly correlated with each other, measuring a single underlying variable, which is as proposed by Fisher and Exline [14], perceived personal responsibility. The CFA results obtained in this study supported the previously identified unidimensional structure, showing adequate fit indices and confirming the good functioning of the Perceived Personal Responsibility Scale.

Regarding the reliability of the Perceived Personal Responsibility Scale by Fisher and Exline [14], high reliability values (between.80 and.84) were found in this study, which coincides with the reliability (Cronbach’s alpha = .83) reported by the original authors. These values are very similar to those reported in other studies that have also used this instrument. For example, Bell et al. [41] obtained a Cronbach’s alpha of.88; Griffin [42] reported a reliability index of.83; and Cornish et al. [43] reported a Cronbach’s alpha of.82. It is noteworthy that all these mentioned studies use exclusive samples of university students from different universities in the United States, with ours being the only one with a non-university sample with heterogeneous characteristics.

As for the evidence of criterion validity, as expected, positive correlations (greater than.40) were found between the Perceived Personal Responsibility Scale and the Genuine Self-Forgiveness and Self-Condemnation subscales. These findings are supported by various studies that have examined the relationship between forgiveness and the assumption of responsibility. More specifically, it has been observed that self-forgiveness does not simply consist of ignoring negative feelings associated with the offense, such as guilt and shame; on the contrary, it involves taking responsibility for one’s actions, reflecting on the mistakes made and working on those feelings [2,3,13,14]. It has also been observed that, on occasion, when a person refuses to work on the negative emotions that arise after assuming responsibility, self-punishing behaviors manifest [3]. This ends up functioning as a means of escape or avoidance of a true self-forgiveness process [3,6,16]. As an alternative, researchers have identified that genuine self-forgiveness must go hand in hand with the process of freeing oneself from self-punitive feelings while still recognizing responsibility [9]. More specifically, self-forgiveness occurs when there is a strong affirmation of values and a restoration of self-esteem [42]. In this sense, accepting personal responsibility for wrongdoing but refusing to address one’s negative emotions will lead to self-punishment rather than self-forgiveness [16].

On the other hand, in this study, a strong and significant negative association was found between the Perceived Personal Responsibility Scale score and Pseudo Self-Forgiveness. In this regard, several authors [10,13] agree that pseudo self-forgiveness is a process that involves not assuming the guilt or responsibility for the harm committed. This lack of assumption of responsibility is often observed when a person excuses themselves for the fault, for example, by placing the responsibility on others. Thus, the individual can restore their self-esteem and avoid a negative self-view, but at the same time, it is associated with low levels of affirmation of violated values, which hinders genuine self-forgiveness [16,42].

It can be concluded that the findings of this study provide sufficient evidence to consolidate the validity of the Perceived Personal Responsibility Scale as an assessment instrument of perceived personal responsibility in different contexts. It is especially relevant to have this validation, which will allow for future comparative research with other cultural contexts and help in a more exhaustive study of perceived personal responsibility in different areas.

Regarding the Desire for Reconciliation Scale by Woodyatt and Wenzel [2], the results of this research have provided relevant information about its psychometric properties, especially concerning its dimensional structure in the Spanish sample. Through EFA, the presence of unifactoriality was identified, indicating that the scale items are correlated and capture a single underlying dimension, which is the desire for reconciliation. To verify the unidimensional structure of this scale, CFA was conducted, and the results confirmed the previously identified unidimensional structure.

In terms of internal consistency, the Desire for Reconciliation Scale ranged from.84 to.88, which is consistent with the original authors’ report (Cronbach’s alpha = .83). In other studies, such as that by Woodyatt & Wenzel [13] the results were similar regarding the instrument’s reliability, with these authors obtaining a Cronbach’s alpha between.90−.94. Bell et al. [41], previously mentioned, obtained a Cronbach’s alpha of.90. Woodyatt et al. [16] reported a reliability index of.84.

However, an additional aspect to consider is the ceiling effect observed in Item 1 of the Desire for Reconciliation Scale, which presented slightly elevated skewness and kurtosis values. This phenomenon reduces the item’s variance and may attenuate item–factor and inter-construct correlations, thereby potentially affecting discriminant validity. In spite of this, the corrected item–total correlation for this item was.45, which falls within the acceptable range reported in the psychometric literature (see S3 Appendix) (≥.30) [22,44]. Although lower than that of the other items, this value indicates that the item still contributes meaningfully to the reliability of the scale, albeit with reduced discriminative capacity. Future studies are encouraged to further monitor the functioning of this item or consider rewording it to enhance its discriminative power.

Regarding the evidence of criterion validity, as expected, positive, significant but weak correlations were found between the Desire for Reconciliation Scale and the Genuine Self-Forgiveness and Self-Condemnation subscales. These findings are consistent with previous studies that explain that in the process of self-forgiveness, a greater desire for reconciliation is observed, especially when the person shows a reaffirmation of violated values [1,16]. Under this approach, the desire for reconciliation ends up functioning as an outcome variable. The observed correlation between the Desire for Reconciliation Scale and the Self-Condemnation subscale is more particular and less studied in the field of self-forgiveness. However, this association is consistent with the fact that, although self-punishment has been shown to be maladaptive, it can sometimes promote personal growth when focused on specific and modifiable aspects of the self, thereby enhancing social connection and self-esteem [45].

At the same time, significant but also weak negative correlations were found with Pseudo Self-Forgiveness. These results suggest that people with a high desire for reconciliation are less likely to excuse themselves from guilt or responsibility without admitting an offense. These data are consistent with previous study results that highlight the importance of recognizing responsibility in self-forgiveness and reconciliation processes.

Similar to the Perceived Personal Responsibility Scale, the results of this study allow us to affirm that the Desire for Reconciliation Scale is a valid instrument for exploring this construct in the Spanish population. The measure we have analyzed proves to be useful for assessing the desire for reconciliation in both personal processes and other contexts, among which we could highlight couples therapy, conflict resolution, peace promotion, restorative justice, among others.

## Conclusions

In summary, this study offers a methodological contribution by validating two brief and theoretically grounded instruments in Spanish. While originally developed in English-speaking settings, both scales demonstrated robust psychometric properties in a culturally and linguistically distinct context. Their application can support further research in forgiveness-related constructs and provide practitioners with reliable tools for assessing key components of self-forgiveness processes in Spanish-speaking populations.

Despite the many contributions of this work, some limitations should be noted. First, although the sample is varied in sociodemographic characteristics, it was recruited through a non-probabilistic sampling strategy, which may limit the generalization of the results. It would be interesting to use more rigorous sampling methods in future studies to increase the external validity of the study. The use of self-report instruments may mean that responses were influenced by the social desirability of the participants.

This study also has notable strengths, among them focusing on two brief and easy-to-apply measurement instruments, relevant for facilitating future research on these emerging concepts, and contributing to advancing knowledge not only in the field of individual psychological processes, such as forgiveness or pseudo-forgiveness but also in other areas, such as those related to conflicts or interpersonal relationships. These instruments show good psychometric properties in the Spanish population and, in this study, a sample with varied characteristics and not exclusively university students, as observed in most studies, was included. Finally, these are two measures that can be applied in clinical treatments, whether individual or collective, focused on working on processes such as self-forgiveness or forgiveness of others, allowing for precise measurement of the impact of such interventions in the Spanish population.

## Supporting information

S1 Appendix Spanish version of the perceived personal responsibility scale (Fisher & Exline, 2006). (PDF)

S2 Appendix Spanish version of the reconciliation desire scale (Woodyatt & Wenzel, 2013). (PDF)

S3 Appendix Corrected item–total correlations for the desire for reconciliation scale. (PDF)

S1 File.
DATA_CFA. Data corresponding to the confirmatory factor analysis of the scales validated in the study.
(XLSX)

S2 File.
DATA_EFA. Data corresponding to the exploratory factor analysis of the scales validated in the study.
(XLSX)
