# Supplementary material for: Validation of the perceived personal responsibility and desire for reconciliation scales in the Spanish population
Source: PLoS One. 2025 Nov 13;20(11):e0336599. doi: 10.1371/journal.pone.0336599 (PMC12614524; doi:10.1371/journal.pone.0336599)
Supplement: S1 Appendix — (PDF) [file pone.0336599.s001.pdf]

Supporting Information

Appendix S1. Spanish version of the Perceived Personal Responsibility Scale (Fisher & Exline, 2006)

	Completamente en desacuerdo									Completamente de acuerdo
Me siento responsable de lo que pasó	1	2	3	4	5	6	7	8	9	10
En realidad, no tuve la culpa de esto (R)	1	2	3	4	5	6	7	8	9	10
Me equivoqué en esa situación	1	2	3	4	5	6	7	8	9	10
Fue claramente mi culpa	1	2	3	4	5	6	7	8	9	10
En realidad, no hice nada malo (R)	1	2	3	4	5	6	7	8	9	10

Note: (R)= Indicate that the item has a reverse score.
