# Supplementary material for: Validation of the perceived personal responsibility and desire for reconciliation scales in the Spanish population
Source: PLoS One. 2025 Nov 13;20(11):e0336599. doi: 10.1371/journal.pone.0336599 (PMC12614524; doi:10.1371/journal.pone.0336599)
Supplement: S2 Appendix — (PDF) [file pone.0336599.s002.pdf]

Supporting Information

Appendix S2. Spanish version of the Reconciliation Desire Scale (Woodyatt & Wenzel, 2013a)

	Totalmente en desacuerdo			Neutral			Totalmente de acuerdo
Solo quiero cosas buenas para esa persona	1	2	3	4	5	6	7
Quiero reconciliarme con esta persona	1	2	3	4	5	6	7
Quiero que nuestra relación mejore	1	2	3	4	5	6	7
Quiero que las cosas vuelvan a estar como estaban antes de que todo esto pasara	1	2	3	4	5	6	7
