# Supplementary material for: Validation of the perceived personal responsibility and desire for reconciliation scales in the Spanish population
Source: PLoS One. 2025 Nov 13;20(11):e0336599. doi: 10.1371/journal.pone.0336599 (PMC12614524; doi:10.1371/journal.pone.0336599)
Supplement: S3 Appendix — (PDF) [file pone.0336599.s003.pdf]

Supporting Information

Appendix S3. Corrected Item–Total Correlations for the Desire for Reconciliation Scale

Item	Corrected Item–Total Correlation	Cronbach's α if Item Deleted
DR 1	.45	.88
DR 2	.81	.73
DR 3	.80	.73
DR 4	.70	.80
